# Emerging Roles of ALK in Immunity and Insights for Immunotherapy

**DOI:** 10.3390/cancers12020426

**Published:** 2020-02-12

**Authors:** Lan Wang, Vivian Wai Yan Lui

**Affiliations:** School of Biomedical Sciences, Faculty of Medicine, The Chinese University of Hong Kong, Hong Kong SAR 999077, China; 1155100020@link.cuhk.edu.hk

**Keywords:** *ALK*-wildtype and aberrations in immunity, ALK-related immunotherapies

## Abstract

Anaplastic lymphoma kinase (ALK) is mostly known for its oncogenic role in several human cancers. Recent evidences clearly indicate new roles of ALK and its genetic aberrations (e.g. gene rearrangements and mutations) in immune evasion, innate and cell-mediated immunity. New ALK-related immunotherapy approaches are demonstrating both preclinical and clinical promises. Here, we provide a timely review on the most updated laboratory and patient-related findings on ALK and immunity, which would grant us important insights for the development of novel ALK immunotherapies for *ALK*-altered cancers.

## 1. Introduction

For the past two decades, anaplastic lymphoma kinase (ALK) is best known for its roles in embryonic and neural development, as well as in human oncogenesis. This receptor tyrosine kinase is now recognized as a clinically druggable target for lung cancer. Until recently, various preclinical and clinical studies have identified new roles of ALK in immune evasion through programmed cell death 1 (PD-L1), as well as in innate, humoral, and T-cell immunities. Furthermore, ALK chimeric antigen receptor (CAR-T) therapy and vaccines are currently under development for *ALK*-altered cancers. Therefore, a better understanding of the emerging roles of ALK in human immune responses may provide novel insights for the development of next generation ALK immunotherapies for *ALK*-altered cancers.

## 2. ALK-Wildtype (ALK-WT) and Its Genetic Aberrations in Human Cancers

The human ALK protein is a 220kDa cell surface receptor tyrosine kinase of the insulin receptor superfamily. Its intracellular kinase domain shares an ~80% sequence homology with that of the leukocyte tyrosine kinase (LTK) [1,2], which is a proto-oncogene known to be involved in immunity or immune-related diseases. The extracellular domain of ALK comprises 2 MAM domains (meprin/A5-protein/PTPmu), a low density lipoprotein class A (LDLa) motif, and a glycine-rich region. ALK can be stimulated by both ligand-dependent or ligand-independent mechanisms. Interestingly, both ALK and LTK share common endogenous ligands, Augmentor α (or FAM150B, ALKAL2) and Augmentor β (or FAM105A, ALKAL1, with relatively higher affinity for LTK) [3,4]. The high sequence homology between ALK and LTK, and their shared ligands support the notion that ALK may have resulted from gene duplication [5].

The role of *ALK* genetic aberrations in human carcinogenesis was widely recognized upon discovery of the *nucleophosmin (NPM)-ALK* gene fusion in anaplastic large cell lymphoma (ALCL) in 1994 [6]. ALCL is a rare subtype of non-Hodgkin lymphoma involving the T lymphocytes. Since then, various gene rearrangements (fusions), mutations, amplification, and alternative splicing events of *ALK* have been identified in several human cancers, including ALCL, inflammatory myofibroblastic tumor (IMT), non-small cell lung cancer (NSCLC), and cancers of the kidney, breast, colon, esophagus, the head and neck, etc. [7,8]. Among these cancers, *ALK* gene fusions were identified to be most common in ALCL (60% cases, mainly *NPM-ALK* fusion) [9], followed by IMT (~50% cases, mainly *TPM3/4-ALK* fusion) [10], NSCLC (~5–7% cases, mainly *EML4-ALK* fusion) [11], and other cancers [8,12,13,14,15] (Table 1). *NPM-ALK* fusion was first demonstrated to be oncogenic in mice, triggering the development of lymphoid malignancy [16]. In fact, functional studies of a wide array of oncogenic *ALK* fusions indicate that various fusion partners of ALK function to promote ALK oligomerization, thus resulting in constitutive activation of the kinase activity of ALK driving cellular transformation.

**Figure 1:**
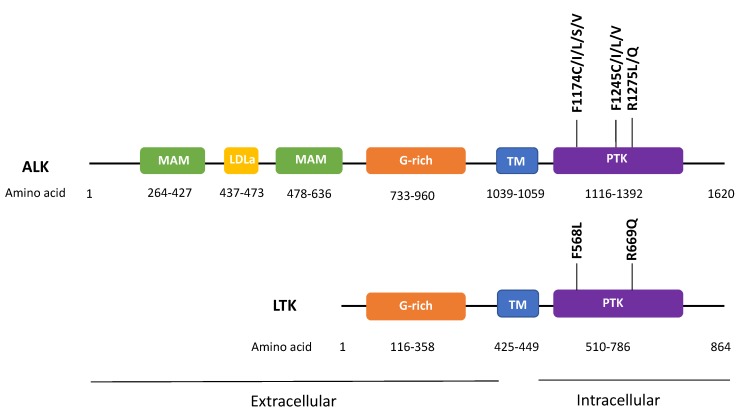
Recurrent oncogenic mutations of *ALK* and corresponding transforming mutations in leukocyte tyrosine kinase (*LTK*). F1174, F1245, and R1275 are three most common mutation sites of *ALK* oncogenic mutations in neuroblastoma; F568L and R669Q in LTK correspond to *ALK* F1174L and R1275Q mutations. meprin/A5-protein/PTPmu (MAM), low density lipoprotein class A (LDLa), G-rich, transmembrane (TM), protein tyrosine kinase (PTK) domains are shown.

In addition to *ALK* fusions, *ALK* point mutations are found in 26 cancer types with an average mutation rate of 1.5%. Functionally, the oncogenic role of *ALK* mutations has only been demonstrated in neuroblastoma, in particular *ALK* F1174C/I/L/S/V, F1245C/I/L/V, and R1275L/Q mutations [17]. Interestingly, mutations of these corresponding amino acid residues on LTK’s kinase domain, namely F568L and R669Q were also found to be transforming in hematopoietic and neuronal cell systems (Figure 1) [1,2]. Recently, various splicing isoforms of *ALK* have also been identified in cancer. Yet, their roles in tumorigenesis remain undefined. Up till now, most of the oncogenic events of *ALK* are known to promote cellular proliferation and survival signaling in cancer models, including the activation of the PI3K, JAK/STAT, and MAPK pathways. In particular, in neuroblastoma, *ALK* events cooperate with *MYCN* amplification to accelerate neuroblastoma development and progression.

## 3. Wildtype ALK in Immune-Privileged Site for Neural Development and Embryogenesis

Though the exact physiological roles of wildtype ALK are not fully known, its tissue expression is largely restricted to the nervous system, an immune-privileged site in the body (i.e., site with peripheral tolerance to self-antigens without eliciting an inflammatory response [18]). In mouse, *ALK* mRNA and protein expressions are restricted to the central and peripheral nervous systems, as well as muscle during embryogenesis [19,20]. After birth, ALK expression is reduced and maintained at a low level thereafter. Similarly, in human, ALK expression is under tight regulation with its expression restricted to the brain with minimal expressions in lung, colon, small intestine, and the testis, as indicated by the expression data of the human protein atlas and several immunohistochemical studies [21]. These expression profiles support the roles of ALK in embryogenesis and neural development. In fact, *Drosophila* ALK (DALK) guides visceral mesoderm differentiation and mediates retinal axon targeting, while the ALK homologue, *T10H9.2* regulates presynaptic differentiation in *Caenorhabditis elegans* [22]. In mammalian systems, ALK drives neurite outgrowth and promotes neuronal differentiation, and *ALK* knockout resulted in hypogonadotropic hypogonadism in male mice due to reduction in Gonadotropin-releasing hormone (GnRH)-positive neurons [23].

## 4. ALK and the STING Innate Immunity

**Figure 2:**
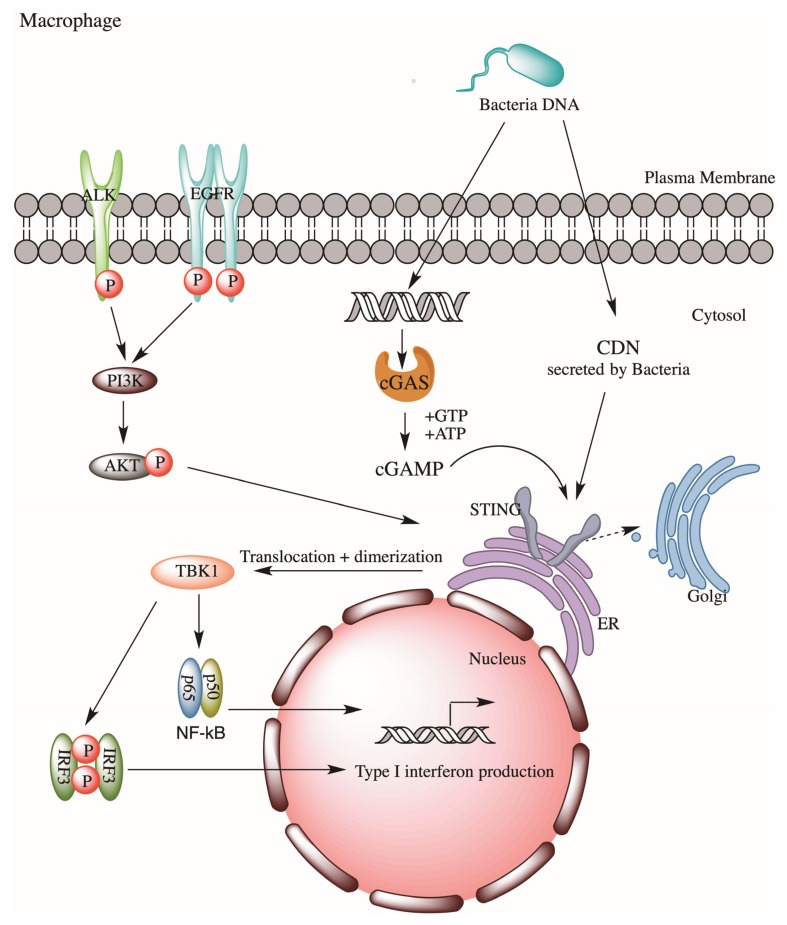
ALK-EGFR-AKT pathway promote STING-dependent innate immune response in macrophage during bacterial sepsis.

The role of ALK in innate immunity is largely unknown. Recently, Zeng et al. reported a novel ALK-STING (the stimulator of interferon genes) pathway in lethal sepsis, first indicating ALK’s involvement in innate immunity against microbial pathogens [24]. STING is a transmembrane adaptor protein on the endoplasmic reticulum which participates in innate responses. In response to exposures to bacterial cyclic dinucleotides (CDNs) or cyclic guanosine monophosphate–adenosine monophosphate (cGAMP) (metabolites derived from DNA of bacteria, virus or damaged cells), STING will stimulate type I interferon (IFN) expression, which will help eliciting a cascade of innate response via activation of NF-κB and interferon regulating factor 3 (IRF3) signaling. Zeng et al. employed a sepsis model and performed a drug library screening and had successfully identified several bioactive compounds as modulators of STING. These included several well-known ALK inhibitors, ceritinib, brigatinib, and AZD3463. Subsequent gene silencing of *ALK* in immortalized mice bone marrow–derived macrophages (iBMDMs) confirmed ALK’s role in STING activation during sepsis. Mechanistically, ALK cooperates with epidermal growth factor receptor (EGFR) to promote AKT phosphorylation, thus STING activation, which then promotes Type I interferon expression via NF-kB and IRF3 (Figure 2). Thus, ALK may represent a potential therapeutic target for lethal sepsis. This ALK-STING axis is later found to be involved in cecal ligation and puncture-induced sepsis in rat models, in which ceritinib-mediated inhibition of ALK significantly reduced sepsis-induced organ injuries and deaths of these septic rats [25]. Such a reduction in deaths was believed to be contributed by the reduction of pro-inflammatory cytokines (e.g., TNF-alpha and IL-6) and sepsis-induced vasculature changes.

## 5. Anti-ALK Antibody Responses against ALK-WT and Fusions in ALCL Patients

Unlike its oncogenic role in cancer, ALK’s potential involvements in humoral as well as cellular immunity were not the center of investigation previously. Interestingly, ALK’s closest homology member, LTK, which is expressed in pre-B and B lymphocytes, is known to be involved in humoral responses. Notably, *LTK*’s gain-of-function polymorphism in human is associated with systemic lupus erythematosus (SLE), a chronic autoimmune disease characterized by autoantibodies production and multi-organ inflammation.

As ALK expression is highly restricted to the nervous system, a highly immune-privileged site, it raises the possibility that throughout development, our human body has limited exposure to ALK protein, thus rendering ALK protein a potential antigen for our immune system. Similarly, tumor-specific ALK fusions or mutants may also be recognized as neoantigens to our body. Therefore, it is plausible that *ALK*-altered cancer cells may have the ability to trigger antibody responses in patients.

In fact, in 2000, Pulford et al. first demonstrated the existence of auto-antibodies against NPM-ALK fusion in ALCL patients. He reasoned that since ALK was absent in normal cells and NPM was abundantly expressed, there was “a strong possibility that NPM-ALK protein would be the target of an immune response” which may account for the paradoxically favorable prognosis of NPM-ALK-positive ALCL patients despite the aggressiveness of the disease [26]. Using an immunocytochemical assay detecting auto-antibodies against NPM-ALK or ALK-WT proteins, they found that 100% of patients (11/11 cases) had anti-NMP-ALK auto-antibodies in their plasma. Most strikingly, auto-antibodies against normal full length ALK-WT was also found in 91% of cases (10/11 patients). No ALK antibody activity was detected in non-cancer normal subjects (*N* = 5). This landmark study demonstrated that humoral immunity against ALK fusion and ALK-WT could be active in ACLC cancer patients. Similar findings were then reported by others [27,28].

Clinically, ALCL with *ALK* gene fusion displays extremely aggressive tumor phenotypes but paradoxically, these patients have noticeably better prognosis than those without *ALK* fusion (with 71% vs. 15% of patients surviving over 10 years) [29]. Findings by Pulford et al. did imply that the production of anti-ALK antibodies by host B cell responses (i.e., humoral responses) may specifically recognize ALK-positive tumor cells and likely help controlling tumor growth. In fact, ALCL patients with high anti-ALK autoantibody had significantly better OS and progression free survival than those with low titers [30]. A negative correlation was identified between the level of ALK autoantibodies and tumor relapse (i.e., the higher the level of ALK auto-antibodies, the lower the risk of disease relapse) [31]. In a kinetic study tracing chemotherapy outcomes and ALK autoantibody levels in ALK-positive pediatric ALCL patients, it was found that patients who maintained higher ALK auto-antibody levels during chemotherapy appeared to have no or less disease relapse [32]. This is consistent with findings in adult ALCL.

In addition to ALCL, ALK auto-antibodies have been detected in ALK-positive NSCLC patients (62%; 13/21 cases), in lymphoma patients with *ALK*-translocation unrelated to *NPM-ALK* (59%; 13/22 cases), in one ALK-positive rhabdomyosarcoma patient, as well as in an *ALK*-WT neuroblastoma patient [28]. Again, normal healthy subjects did not carry any ALK auto-antibodies. Thus, anti-ALK antibody response was not limited to ALCL, but also found in cancer patients bearing either ALK fusion or ALK-WT protein expression. In NSCLC, 17% of ALK-positive NSCLC patients had strong anti-ALK antibody response in their sera, while such an autoantibody response against ALK was absent in ALK-negative NSCLC patients [33]. Table 2 summaries all ALK auto-antibody reported thus far. These clinical findings support Pulford’s hypothesis that since ALK was absent in normal cells, antibody responses against ALK fusion proteins or even ALK-WT would be possible. The fact that ALK auto-antibody cannot be detected in normal subjects may imply that *ALK*-altered cancer patients may have an immune-activated, or perhaps, an inflammatory state when compared to normal subjects. Thus, boosting a patient’s existing immunity against ALK or ALK aberrations by vaccination may serve as an effective approach for the treatment of ALK-positive ALCL and other *ALK*-rearranged cancers.

## 6. Cytotoxic T-Cell Responses against ALK with Epitope Identification

In addition to initiating humoral responses, neoantigens or tumor specific antigens can potentially induce cytotoxic T-cell (CTL) responses for eradication of tumor cells. In 2002, Passoni et al. postulated that ALK could be “an ideal tumor antigen” due to its restricted expression in the “immune privileged” nervous system in which antigens were not presented to the immune system (note: this is true under normal conditions when the nervous system is not compromised by diseases such as neurodegenerative diseases) [34]. This simply means that if anti-ALK-specific CTL precursors exist in the body, they may not be eliminated by the thymus by mechanism of self-tolerance. Passoni et al. further reasoned that since in ALCL, the strong NPM promoter should drive high levels of NPM-ALK fusion protein expression and proteasome processing, high levels of ALK epitopes would likely be exposed to our immune system to trigger tumor-specific CTL responses. In fact, they screened 9 ALK peptides for their binding activities to the human leukocyte antigen (HLA)-A*0201 molecules in vitro (an essential step for antigen presentation), tested their in vivo immunogenicity in mice, as well as their abilities to stimulate ALK-specific CTL responses in vitro using human peripheral blood lymphocytes (PBLs). With such a screening strategy, they identified two immunogenic ALK epitopes that could elicit CTL responses in vitro, in vivo and with human PBLs. These two immunogenic ALK epitopes represented peptide sequences derived from ALK’s kinase domain, p280–89 (SLAMLDLLHV) and p375–86 (GVLLWEIFSL). Most strikingly, such peptides were able to induce specific CTL activity, causing specific lysis of HLA-A-matched ALK-positive ALCL cells, and neuroblastoma cells in vitro. A subsequent study by Singh et al. further narrowed down the immunogenic region for T-cell responses to peptides between p327-p370 of NPM-ALK. In addition to HLA-A-restricted T-cell response, HLA-B and HLA-C allele-restricted T-cell responses against NPM-ALK have also been recently identified in 40% of pediatric or adolescent NPM-ALK ALCL patients [35].

In another study, Passoni et al. further determined the natural existence of anti-ALK CD8+ precursor repertoire in ALCL patients by analyzing both the frequency of circulating ALK CTL precursors and their functional status in ALCL patients vs. healthy subjects [36]. Using a major histocompatibility complex (MHC) tetrameric assay to assess the presence of T-cells with receptors matching with the ALK peptide p280-89 in the peripheral blood mononuclear cells (PBMC) of tested subjects, high levels of anti-ALK CD8+ repertoire were detected in both ALK-positive ALCL patients and healthy subjects. Importantly, functional analyses of circulating anti-ALK CD8+T lymphocytes from ALCL patients identified the presence of high levels of both effector and central memory T-cells in these patients. Whereas in healthy subjects, the anti-ALK CD8+T-cells were all naïve (lacking effector nor memory T-cells). Using an ELISPOT assay (which measures IFN-γ as a functional readout of CTL cytolytic activity), they further showed that these circulating anti-ALK CTLs from ALK-positive ALCL patients were cytolytically active with low activation threshold for effective lysis of ALK-positive lymphoma cells, conclusive of functional memory T-cells in ALCL patients. It was rationalized that the natural existence of a memory T-cell activity in ALCL patients would ideally favor the mounting of potent secondary immune responses in these patients by simple ALK-peptide re-stimulation with a vaccination approach. This landmark study lays an important foundation for vaccine-based therapy for ALK-positive cancers.

**Figure 3:**
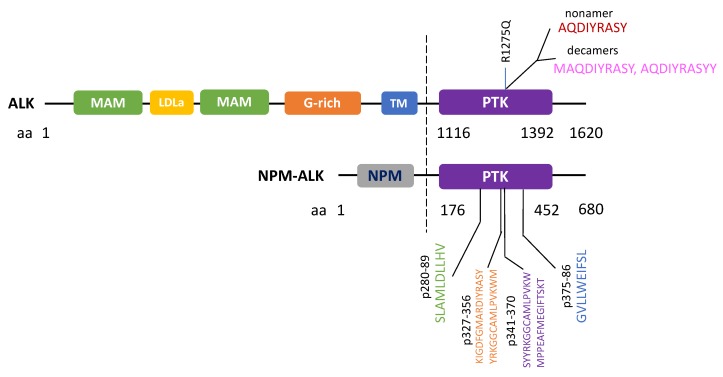
ALK Immunogenic peptides known to trigger cytotoxic T-cell responses.

In addition to functional approaches, a bioinformatics approach called Prediction of T Cell Epitopes for Cancer Therapy (ProTECT) has been recently developed to identify ALK neoepitopes from recurrent *ALK* R1275Q mutation [37]. ProTECT allows the prediction of high affinity binders (epitopes) to common HLA alleles, with which a nonamer (AQDIYRASY) and two decamer sequences of ALK (MAQDIYRASY and AQDIYRASYY) with high affinities to HLA-B*15:01 were identified. These peptides were then experimentally confirmed to be able to raise CD8+ T-cell recognition using patient PBMC with matched HLA-alleles [38]. Overall, the above studies showed that immunogenic ALK epitopes could be identified against ALK mutant as well as ALK-fusion cancers. All reported ALK immunogenic peptides which were known to trigger CTL responses are summarized in Figure 3.

## 7. ALK in Inflammasome Activation and Cytokines

ALK has recently been demonstrated to regulate NLR family pyrin domain 3 (NLRP3) inflammasome activation, the most common form of inflammasome known thus far. In general, inflammasomes can regulate host immune responses and cause human diseases by inducing pyroptosis (proinflammatory programmed cell death) and the release of cytokines such as IL-1β and IL-18. ALK has been shown to regulate and promote activation of NLRP3 inflammasome in macrophages [38]. Using *ALK* siRNA, and ALK inhibitors, ceritinib and lorlatinib, Zhang et al. showed that ALK was capable of regulating both the priming and the sensing steps of pyroptotic cell death in mouse bone marrow-derived macrophages [38]. In the priming step of NLRP3 inflammasome formation, ALK mediates NF-kB activation, which then activates LPS-induced *NLRP3* transcription. While in the sensing step, ALK mediates lipid peroxidation to stimulate NLRP3 activation, pyroptotic cell death of macrophages, and the release of IL-1β. Thus, ALK targeting might represent a novel strategy to treat NLRP3 inflammasome-mediated diseases.

In relation to the above findings, at least in two of the cancer types that are commonly affected by ALK fusions are well-recognized to be highly inflammatory in nature with increased cytokine levels found in patients. These are ALCL and IMT. ALCL can be manifested either in the skin (primary cutaneous ALCL), systematically in lymph nodes or in other organs (systemic ALCL). Whilst IMT involves chronic inflammatory infiltrates by eosinophils, lymphocytes and plasma cells. In these inflammatory diseases, cytokines, the key mediators regulating immune cell activities are potentially involved in disease pathogenesis.

In NPM-ALK-positive ALCL cells, IL-22 and IL-22R1 autocrine signaling has been shown to be increased [39]. Ectopic expression of the *NPM-ALK* fusion gene could induce IL-22R1 expression in ALCL cells, and IL-22 stimulation was found to activate STAT3 oncogenic signaling via this NPM-ALK/IL-22R1 axis. In fact, IL-22 neutralizing antibodies could efficiently reduce the clonogenicity of NPM-ALK-positive ALCL cells, concluding the functional importance of IL-22 autocrine signaling in ALCL tumorigenesis. Importantly, it was found that in ALCL patients treated with EPOCH (etoposide, prednisone, vincristine, cyclophosphamide, and doxorubicin) chemotherapy, those patients with undetectable serum IL-22 levels demonstrated complete remission or no relapse. Thus, serum IL-22 levels may predict treatment responses in ALCL patients. In addition to IL-22, various pro-inflammatory cytokines and their receptors were found to be significantly increased in ALCL patients. These include: IL-1β, IL-2, soluble IL-2 receptor (sIL-2R), IL-7, IL-8, IL-9, TNF-α, TNFSF10, TNFSF13, hepatocyte growth factor (HGF), CD30, and TRAP1 [40,41,42]. In particular, neutralizing antibody against IL-9 was found to inhibit ALCL cell survival and clonogenicity in vitro. Thus IL-9 and IL-22 neutralizing antibody approaches should be further developed for their potential therapeutic activity for ALCL. Lastly, serum IL-6 level was also found to be prognostic for poor survival in ALK-positive ALCL [43]. In IMT patients, IL-6 has also been reported to be elevated [44], however its role in IMT remains undefined.

## 8. ALK Aberrations and Immune Evasion Signaling

Although *ALK* gene fusions are known to trigger both the humoral and CTL responses in ALCL patients, there are recent reports showing that *ALK* fusions can also induce the expressions of an important immune checkpoint protein, programmed cell death 1 (PD-L1). Such an ALK-induced PD-L1 upregulation may potentially confer an immunosuppressive tumor microenvironment contributing to tolerance and immune evasion in cancer. This phenomenon of PD-L1 upregulation has been reported in *NPM-ALK*-rearranged T-cell lymphoma [45] and *ALK*-rearranged NSCLC [46,47,48].

Marzec et al. first reported that in *NPM-ALK*-rearranged T-cell lymphoma cell models, NPM-ALK fusion could induce PD-L1 expression via direct transcriptional activation by STAT3 at the *PD-L1* promoter [45]. Furthermore, NPM-ALK fusion, through STAT3 activation, could also upregulate the expression of other immunosuppressive signaling, including IL-10 and TGFβ. These findings implicate that the oncogenic *NPM-ALK* fusion gene not only could directly activate STAT3 oncogenic signaling, but also potentially grant these fusion-bearing cancer cells additional immune evasion ability by upregulating multiple immunosuppressive mechanisms. Another study also provided clinical evidences supportive of the STAT3/PD-L1 axis in ACLC patient samples that the ALK-positive status was associated with PD-L1 expression and STAT3 activation [49].

PD-L1 was found to be more frequently expressed in *ALK*-rearranged NSCLC (60% cases) than in *EGFR*/*KRAS*/*ALK*-WT NSCLC (~20%) [46]. Ota et al. recently showed that *ALK*-rearranged NSCLC patient tumors expressed a significantly higher level of PD-L1 than patients with *ALK*-WT tumors [47]. Screening of surface PD-L1 protein expression, as well as *PD-L1* mRNA expression in a panel of *ALK*-rearranged vs. -WT NSCLC cell lines also confirmed the presence of a higher level of PD-L1 in *ALK*-rearranged NSCLC cells. Importantly, two independent studies showed that ALK inhibitors, alectinib and TAE684, as well as *ALK* siRNA could effective inhibit the expression of EML4-ALK-induced PD-L1 expression in NSCLC cell models, confirming a positive role of ALK activity on PD-L1 induction in NSCLC [47,48].

In fact, these findings are consistent with the notably low immune infiltrating levels in *ALK*-rearranged NSCLC tumors [50], supportive of the notion that *ALK*-altered cancers can be immunosuppressive in their microenvironments. In line with these observations, Hong et al. successfully demonstrated that anti-PD-1/PD-L1 antibodies could potentially be therapeutic in *ALK*-rearranged NSCLC [48]. Using a co-culture system with tumor cells and cytokine-Induced Killer (CIK) cells, they further demonstrated that anti-PD-1 antibody treatment could reduce the death of T-cells, activate T-cell immunity with resultant growth inhibition of *EML-ALK*-rearranged NSCLC by CIK cells. These findings implicate the potential beneficial effects of PD-L1 targeting in *ALK*-rearranged NSCLC. Clinically, patients with *ALK*-rearranged NSCLC do develop resistance to ALK inhibitors with reasons related to secondary *ALK* gene mutations or other bypass signaling activation. In fact, a recent study showed that PD-L1 expression was increased upon development of crizotinib resistance in ALK-positive NSCLC cell lines and patient tumors, suggesting the potential involvement of PD-L1 increases during disease progression and resistance to therapy [51].

With regard to the mechanisms underlying *ALK* fusion-induced PD-L1 in NSCLC, several studies demonstrated the involvement of PI3K and MAPK activation [47], as well as the Hippo pathway [52]. Through a kinome-wide screen of modulators of the Hippo pathway, Nouri et al. identified ALK as an inducer of the new ALK-LATS-YAP-PD-L1 axis in NSCLC cells expressing EML4-ALK. It remains to be examined if this new ALK-LATS-YAP-PD-L1 axis may also exist in other cancer types and exert its immunosuppressive effect in cancers other than NSCLC.

Though ALK-PD-L1 emerges as one major immune evasion mechanism studied thus far, a recent study revealed a plausible regulation of antigen presentation machinery, the HLA system, by ALK. In NPM-ALK-positive ALCL cell models, ALK inhibitor treatments (crizotinib, alectinib) were found to significantly increase the cell surface expression of HLA-A/B/C [53]. Thus, ALK kinase activity appears to mediate suppression of HLA proteins, a key antigen-presentation machinery in human. Therefore, it will be interesting to examine if ALK inhibitors could be further explored as potential adjuvants to promote or enhance T-cell based immunotherapies by upregulating HLA expressions in cancer.

## 9. Immunotherapy Developments for ALK-Associated Cancers

Current drug therapies for ALK-positive cancers are mainly precision treatments with ALK inhibitors (crizotinib, ceritinib, alectinib, brigatinib, lorlatinib). Most of these inhibitors will eventually lead to the development of drug resistance in patients. With the emerging immune-related findings in *ALK*-altered cancers, various preclinical and clinical efforts are ongoing to identify novel ALK-related immunotherapies with potentially better or more sustained therapeutic effects or even cure of these cancers.

### 9.1. Immune Checkpoint Inhibitors in ALK-Positive Cancers

As mentioned above, *ALK* aberrations can induce PD-L1 upregulation in *ALK*-rearranged-ALCL and -NSCLC. These new findings suggest the potential use of anti-PD-L1 therapy for these cancers. Recently, a refractory ALK-positive ALCL patient has demonstrated prolonged responses to the PD-1 inhibitor, nivolumab, with dramatic reduction of physical pain and tumor size [54]. On the other hand, isolated reports noted that some NSCLC patients with *ALK* rearrangements or *EGFR* mutations had low response rates to PD-1/PD-L1 inhibitors, possibly due to low PD-L1 expression and low levels of infiltrating CD8+T lymphocytes in tumors [55]. Currently, ongoing clinical trials are carefully examining the potential clinical benefits of PD-1/PD-L1 checkpoint inhibitors and their safety in patients with *ALK*-rearranged cancers. In particular, combinations of ALK inhibitors with PD-1/PD-L1 therapies have shown early promises in advanced ALK-positive NSCLC, but yet drawing attention to the right drug combinations with good safety profiles. For example, results from the CheckMate 370 study (NCT02574078) study showed that combination of crizotinib, the first generation of ALK inhibitor, with nivolumab at the respective doses of 250mg twice daily and 240mg every two weeks appeared to exhibit some severe toxicities in advanced *ALK*-rearranged NSCLC patients [56]. Importantly, studies on ceritinib/nivolumab combination (NCT02393625) showed promising clinical activities in patients [57], and lorlatinib with avelumab (anti-PD-L1 antibody; NCT02584634) showed promising antitumor activities with acceptable safety profiles than crizotinib/avelumab combination [58]. Similarly, alectinib/atezolizumab combination (NCT02013219) [59] also demonstrated promising anti-tumor activity in patients with acceptable safety profiles. These results seem to support the use of combinations with newer generations of ALK inhibitors with anti-PD-1/PD-L1 therapies in advanced *ALK*-rearranged NSCLC.

In addition to *ALK*-rearrangements, would other *ALK* aberrations e.g., mutations be able to alter the PD-L1 axis, thus rendering other *ALK*-altered cancers amendable to anti-PD-L1 therapy? Recently, Lombardo et al. showed that *ALK* mutations (and ALK activation) could regulate PD-L1 expression in neuroblastoma cell models using a computational approach predicting potential intracellular events/factors that may influence PD-L1 expression in cancer. This in silico approach attempts to efficiently examine potential interactions of *ALK* aberrations and PD-L1 signaling in human cancers. If validated in clinical settings, this in silico approach may impact the design of immunotherapy regimens for *ALK*-altered cancers.

### 9.2. ALK Neoantigens and ALK Vaccines

T-cell-mediated immune response against tumor neoantigens can effective eradicate tumor cells in patients. In melanoma patients, personalized neoantigen vaccination has been shown to protect against cancer recurrences [60], as neoantigen vaccination can elicit specific T-cell responses against tumor cells.

ALK is a promising neoantigen which can be harnessed for vaccination therapy as T-cell responses can be triggered by *ALK* aberrations as discussed above. Two ALK peptides (SLAMLDLLHV, GVLLWEIFSL) located on the ALK kinase domain have already been shown to be immunogenic CD8+T-cell epitopes (HLA-A*0201-restricted) in ALK-positive ALCL patients [34]. Furthermore, these peptides could trigger specific anti-ALK CLTs to lyses ALK-positive ALCL cells and neuroblastoma cells in an HLA-matched manner. In an ALK-positive lymphoma mouse model, DNA vaccines with plasmids encoding part of the ALK cytoplasmic domain effectively prevented the development of systemic and local lymphoma via specific CD8+T-cell cytotoxicity and interferon-γ response. In ALCL, combination of chemotherapy and *ALK* DNA vaccine could also prevent in vivo tumor growth and increased survival of lymphoma-bearing mice [61]. This *ALK* DNA vaccine also resulted in a 60% reduction in tumor number with survival doubling in ALK-positive NSCLC mouse models [50]. Another DNA plasmid-based *ALK* vaccine was also found to be effective in mounting specific anti-ALK immune response in ALK-positive NSCLC mouse models [62]. With the ProTECT bioinformatics approach, it is possible that more ALK epitopes/peptides can be developed into therapeutic vaccines even for other cancers bearing likely immunogenic *ALK* mutations. With additional safety studies in large animals, it is likely that *ALK* vaccines can be assessed in clinical settings in near future.

### 9.3. Anti-ALK Antibody Therapy

Antagonistic or neutralizing antibodies are useful therapies for many human malignancies. These include the use of cetuximab against EGFR in head and neck cancer, and traustuzumab against HER2 in breast cancer, etc. As ALK is a cell surface receptor, it can be potentially druggable with such a neutralizing antibody approach. In fact, anti-ALK antibody may have limited toxicity against normal human tissues as ALK expression is largely restricted to the well-protected nervous system. Efforts to develop anti-ALK antibodies are ongoing. Several monoclonal antibodies targeting ALK extracellular domain have been shown to effectively inhibit ligand-dependent activation of ALK with cell growth inhibition in vitro [63].

A single-chain antibody approach (svFC) targeting ALK’s ligand-binding domain has also been shown to be highly effective in inhibiting the in vivo growth of ALK-positive glioblastoma. Stylianou et al. employed a tetracycline-inducible system to express a secretable anti-ALK svFC in U87MG glioblastoma cells. They found that upon doxycycline induction, the anti-ALK svFC-expressing tumors had a significant reduction in tumor size as compared with tumors with no induced expression of the antibody, first demonstrating the therapeutic efficacy of the anti-ALK antibody therapy approach in principle [64]. Further, in human neuroblastoma-derived cell lines with either WT or mutated *ALK* (F1174L, R1275Q), antagonistic antibody against ALK’s extracellular domain resulted in specific inhibition of neuroblastoma cell growth with antibody-dependent cellular toxicity in vitro [65]. These promising preclinical findings provide strong rationale for future clinical testing of neutralizing ALK antibodies in patients.

Furthermore, since *ALK*-rearranged ALCL patients appear to have active humoral immunity generating endogenous auto-antibodies against ALK, future studies should examine the potential therapeutic effects of ALK-related vaccines in these patients.

### 9.4. CAR-T Cell Therapy Targeting ALK

Chimeric antigen receptor (CAR)-T cells are therapeutic T-cells engineered to recognize specific cell surface protein on cancer cells, allowing effective eradication of tumor cells. Recently, CAR-T therapy has rapidly emerged as an effective therapy for acute lymphoblastic leukemia and advanced lymphomas.

Residing on the cell surface, oncogenic ALK protein has been recently examined as a potential CAR-T target for ALK-positive cancers. Walker et al. designed ALK CAR-T cells with CARs of single chain variable fragments against ALK extracellular domain [66]. These ALK CAR-T cells can lyse ALK-positive neuroblastoma cells in co-cultures. The CAR-T cell function was found to be dependent on the density of ALK antigen and the expression level of CARs. Yet, one has to be reminded that for CAR-T therapy, the cell surface portion of the ALK fusion events should be the CAR-T target. In most *ALK*-rearranged cancers, the actual extracellular portions are the fusion partners of *ALK*, while the ALK kinase domain usually resides intracellularly. Therefore, ALK CAR-T approach should also consider targeting the extracellular fusion partners of ALK such as EML4 or NPM.

## 10. Conclusions

ALK is a therapeutic target for multiple *ALK*-altered cancers. Our recent understanding of ALK in innate and cell-mediated immune responses, as well as PD-L1 regulation starts to provide initial clues for future design of ALK immunotherapy. Early preclinical and clinical successes discussed above will likely drive more efforts towards the development of various ALK immunotherapies for *ALK*-altered cancers.

## Figures and Tables

**Table 1:** Common anaplastic lymphoma kinase (*ALK*) gene fusions found in various cancers.

Cancer Type	Frequency of *ALK* Fusions	Major *ALK* Gene Fusions	Chromosomal Translocation	References
**ALCL**	~60%	*NPM-ALK* (~75%)	t(2;5)(p23;q35)	[9]
		*TPM3-ALK* (~18%)	t(1;2)(q25;p23)	
**IMT**	~50%	*TPM3-ALK* (~50%)	t(1;2)(q25;p23)	[10]
		*TPM4-ALK* (~50%)	t(2;19)(p23;p13)	
**NSCLC**	~5%–7%	*EML4-ALK* (~75%)	inv(2)(p21;p23)	[11]
**DLBCL**	~1%	*CLTC-ALK*	t(2;17)(p23;q23)	[12]
**Colorectal Cancer**	~1%	*EML4-ALK*	inv(2)(p21;p23)	[8]
**Breast Cancer**	~1%	*EML4-ALK*	inv(2)(p21;p23)	[8]
**Esophageal Cancer**	ND	*TPM4-ALK*	t(2;19)(p23;p13)	[13]
**Renal Cancer**	ND	*VCL-ALK*	t(2;10)(p23;q22)	[14]
**Ovarian Cancer**	ND	*FN1-ALK*	t(2;2)(p23;q35)	[15]

Keys: ND: not determined; DLBCL: Diffuse large B-cell lymphoma.

**Table 2:** Auto-antibody responses reported against ALK fusions in cancer.

Cancer Types	*ALK* Gene Fusions	Antibody Detected	Detection Rate	Level of Antibody Titer		References
				High	Low	
**ALK + ALCL**	*NPM-ALK*	Anti-NPM-ALK antibody	100%	ND	ND	[26]
			(11/11 pts)			
		Anti-ALK-WT antibody	91%	ND	ND	
			(10/11 pts)			
	*NPM-ALK*	Anti-NPM-ALK antibody	100%	78%	22%	[27]
			(9/9 pts)	(7 pts)	(2 pts)	
	*NPM-ALK*	Anti-NPM-ALK antibody	89%	60%	40%	[32]
			(25/28 pts)	(15 pts)	(10 pts)	
	*NPM-ALK*	Anti-NPM-ALK antibody	92%	78%	22%	[29]
			(87/95 pts)	(68 pts)	(19 pts)	
	*NPM-ALK*	Anti-NPM-ALK antibody	96%	72%	28%	[30]
			(123/128 pts)	(89 pts)	(34 pts)	
	*X-ALK* but not *NPM-ALK*	Anti-ALK antibody	59%	75%	25%	[28]
			(13/22 pts)	(9 pts)	(3 pts)	
**ALK + NSCLC**	*EML4-ALK*	Anti-EML4-ALK antibody	62%	23%	77%	[28]
			(13/21 pts)	(3 pts)	(10 pts)	
	*EML4-ALK*	Anti-EML4-ALK antibody	17%	100%	Nil	[33]
			(9/53 pts)	(9 pts)		

Keys: *X-ALK* indicates *ALK* fusions with various fusion partners; Pts: Patients; ND: not determined; High antibody titer: > 1/750; Low antibody titer: 0 ≤ 1/750 (antibody titer refers to dilution reported by the original publications).
